# Physiologically relevant coculture model for oral microbial-host interactions

**DOI:** 10.1038/s41368-025-00365-9

**Published:** 2025-05-27

**Authors:** Zeyang Pang, Nicole M. Cady, Lujia Cen, Thomas M. Schmidt, Xuesong He, Jiahe Li

**Affiliations:** 1 Department of Biomedical Engineering, College of Engineering and School of Medicine, University of Michigan, Ann Arbor, MI USA; 2 Department of Microbiology and Immunology, University of Michigan Medical School, Ann Arbor, MI USA; 3 Department of Microbiology, The ADA Forsyth Institute, Somerville, MA USA; 4 Department of Internal Medicine, University of Michigan, Ann Arbor, MI USA; 5 Department of Ecology and Evolutionary Biology, University of Michigan, Ann Arbor, MI USA

**Keywords:** Microbiology techniques, Periodontitis, Cellular microbiology

## Abstract

Understanding microbial-host interactions in the oral cavity is essential for elucidating oral disease pathogenesis and its systemic implications. In vitro bacteria-host cell coculture models have enabled fundamental studies to characterize bacterial infection and host responses in a reductionist yet reproducible manner. However, existing in vitro coculture models fail to establish conditions that are suitable for the growth of both mammalian cells and anaerobes, thereby hindering a comprehensive understanding of their interactions. Here, we present an asymmetric gas coculture system that simulates the oral microenvironment by maintaining distinct normoxic and anaerobic conditions for gingival epithelial cells and anaerobic bacteria, respectively. Using a key oral pathobiont, *Fusobacterium nucleatum*, as the primary test bed, we demonstrate that the system preserves bacterial viability and supports the integrity of telomerase-immortalized gingival keratinocytes. Compared to conventional models, this system enhanced bacterial invasion, elevated intracellular bacterial loads, and elicited more robust host pro-inflammatory responses, including increased secretion of CXCL10, IL-6, and IL-8. In addition, the model enabled precise evaluation of antibiotic efficacy against intracellular pathogens. Finally, we validate the ability of the asymmetric system to support the proliferation of a more oxygen-sensitive oral pathobiont, *Porphyromonas gingivalis*. These results underscore the utility of this coculture platform for studying oral microbial pathogenesis and screening therapeutics, offering a physiologically relevant approach to advance oral and systemic health research.

## Introduction

The oral cavity is a complex ecosystem that hosts many different species of microorganisms.^1^ Oral microbes play a crucial role in the development of oral chronic inflammation diseases such as gingivitis and periodontal disease.^2,3^ It has become increasingly appreciated that microbial-host cell interaction affects local immune responses in the oral cavity and may also profoundly affect systemic health through inflammatory pathways.^4,5^ To maintain homeostasis, the gingival epithelium provides a physical and biological barrier responsible for host defense yet is frequently challenged by pathogenic bacteria. Therefore, understanding the interaction between oral bacteria and host tissues is essential for both local oral health and the prevention of systemic diseases. To understand the exact role of oral microorganisms in diseases, the coculture system, which allows for the simultaneous cultivation of bacteria and host cells, has become an essential tool in this field. By simulating the interaction between anaerobes and host cells, we can better understand the pathogenic mechanisms of these microorganisms in certain disease models and even explore their potential role in systemic diseases.^6^ Therefore, studying the coculture of oral microorganisms and host cells is significant in promoting a scientific understanding of the relationship between the oral cavity and systemic health.

However, there are significant limitations in current bacterial-host cell coculture systems, particularly when involving anaerobic bacteria.^7^ In traditional cell culture systems, anaerobic bacteria are typically introduced directly into the normoxic culture medium, which presents several challenges. First, anaerobic bacteria often struggle to maintain viability in these environments due to the presence of oxygen.^8^ This significantly impairs their growth and metabolism, limiting the duration of studies to less than 12 hours.^9^ Second, the oral environment naturally contains distinct normoxic and anaerobic conditions, exposing cells and bacteria to varying oxygen levels.^10^ Without replicating these differences in oxygen concentration, it is challenging to accurately simulate the physical and chemical properties of the oral cavity, making coculture systems less representative of actual physiological conditions.^11^ Lastly, the conventional method fails to effectively simulate the complex process by which bacteria invade host cells, limiting the relevance and accuracy of the long-term models in reflecting in vivo conditions.^9^ As a result, these limitations hinder the ability to fully understand oral bacterial pathogenesis and necessitate the development of improved coculture techniques that better simulate the natural interactions between anaerobic bacteria and host cells. To date, in vitro devices to mimic the gingival pockets are complex and do not necessarily allow for facile characterization of microbe-host interactions.^12,13^

**Fig. 1:**
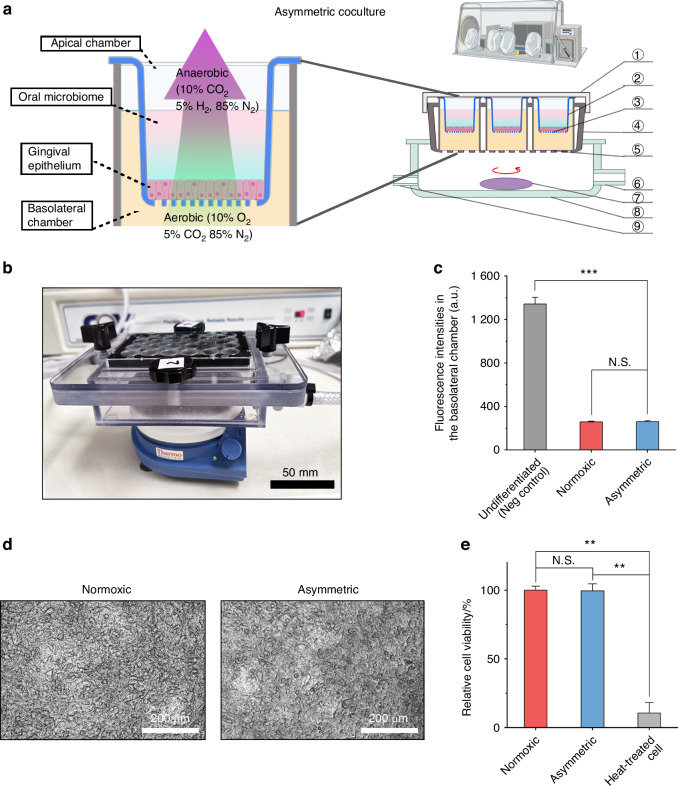
Development of an asymmetric gas coculture system utilizing an immortalized gingival epithelial cell line as a barrier. **a** A schematic illustration depicting the gas composition within the apical and basolateral chambers of an asymmetric coculture setup housed in a vinyl anaerobic chamber, as well as the overall composition of the entire asymmetric coculture system. (Left) Input gas containing 10% oxygen enters the basolateral chamber to support cell monolayer respiration, progressively depleting the oxygen. Concurrently, the apical chamber maintains an anaerobic environment that closely replicates the surrounding anaerobic conditions, thereby ensuring the optimal growth of anaerobic bacteria. (Right) The plate cap ① covers the gas-permeable plate ④, preventing contamination and leakage. The apical chamber of the Transwell inserts ② is filled with coculture medium (CCM, see Table S1), which supports the stable growth of the cell monolayer ③ and oral anaerobes. The lower compartment (i.e., the basolateral compartment) of the Transwell insert is filled with cell culture medium. The gas-permeable membrane ⑤ at the bottom ensures unidirectional oxygen diffusion. Basolateral gas flow containing 10% oxygen enters through the gas inlet ⑨, spreading evenly through the asymmetric coculture chamber ⑧ with a magnetic stirrer ⑦. Exhaust gas is discharged through the gas outlet ⑥, completing the system’s airflow.^6^ The figure was created using BioRender. **b** A physical picture of the asymmetric coculture chamber. **c** Comparison of fluorescence intensities of FITC-dextran in the basolateral chamber at 24 h after adding FITC-dextran to the apical chamber of Transwells containing TIGK monolayers. Undifferentiated (negative control) and differentiated TIGKs under the normoxic culture conditions (referred to as “normoxic”) were compared to differentiated TIGKs under the asymmetric culture condition (referred to as “asymmetric”). The background fluorescence intensity of the blank culture medium was subtracted for each condition. Undifferentiated TIGK monolayer cultured under the normoxic condition before switching to a differentiation medium containing Ca^2+^ serves as the negative control (N.S., *P* > 0.05, *** *P* < 0.001, *n* = 2 technical replicates, *N* = 3 biological repeats). **d** The morphology of TIGK monolayers in Transwell inserts maintained in a cell culture incubator under normoxic conditions or cultured in an asymmetric coculture chamber for 24 h. The collagen coating is known to affect bright field imaging due to the presence of the collagen fibers, which can obscure fine details of the cells or structures being observed compared to uncoated surface.^62^ **e** Comparison of cell viability in TIGK monolayers cultured under normoxic and asymmetric culture conditions. Heat-treated cells are the negative control (N.S., *P* > 0.05, ** *P* < 0.01, *n* = 3, *N* = 3)

To address these gaps, we propose an asymmetric gas coculture system to support the viability of both the cell monolayer and anaerobic bacteria, which allows for studying bacterial invasion and infection under physiologically relevant conditions (Fig. 1a). Although this setup has been employed for interrogating the gut microbe-host interactions in vitro, to the best of our knowledge, a similar system has not been used for characterizing the interplay between oral microbes and the host.^6^ As a proof-of-concept, we employed both *Fusobacterium nucleatum* (*Fn*) and *Porphyromonas gingivalis* (*Pg*), two key oral pathobionts, to demonstrate the utility and advantages of our system. Both pathogens are of particular interest due to their association with periodontal and systemic diseases.^14–18^ We demonstrate that our coculture system outperforms traditional platforms by sustaining the viability of both the cell monolayer and the anaerobic bacteria, allowing oral anaerobes to thrive under anaerobic conditions while maintaining oral epithelial cells at physiological oxygen levels. This approach holds the potential to advance our understanding of microbial pathogenesis in oral and systemic diseases and serves as a valuable platform for drug screening and therapeutic development.

## Results

### The asymmetric gas chamber maintains the viability of telomerase-immortalized gingival keratinocytes (TIGKs)

The oral cavity is a dynamic environment characterized by varying oxygen levels across different regions, influenced by factors such as proximity to oxygen-rich surfaces (e.g., saliva and open air) and the presence of oxygen-consuming bacterial communities. At the bacterial-host interface, the differential oxygen levels become even more pronounced due to the metabolic activity of both the host cells and the microbial community.^19,20^ Traditional coculture systems, however, subject host cells and oral anaerobes to uniform, oxygen-rich (normoxic) conditions, failing to accurately mimic the physiologically relevant microenvironment of the oral cavity. To overcome these limitations, we developed an innovative asymmetric gas chamber system that replicates the contrasting aerobic and anaerobic conditions observed in the oral cavity. Our experimental setup utilizes telomerase-immortalized gingival keratinocytes (TIGKs) cultured on Transwell inserts (Fig. 1a and Supporting Video). TIGKs were chosen for their biological similarity to primary human gingival epithelial cells, making them ideal for in vitro studies.^21^ The apical compartment was maintained under anaerobic conditions, while the basolateral chamber was supplied with a 10% oxygen gas mixture, simulating two distinct culture environments (Fig. 1a). This setup allows for the simultaneous cultivation of host cells and anaerobic bacteria under physiologically relevant conditions, facilitating more accurate studies of microbial-host interactions.

Before transferring TIGKs into the asymmetric gas coculture chamber, the cells—exhibiting a polygonal shape, close packing, and large, round nuclei—were first seeded in Transwell inserts in Dermal Cell Basal Medium (Fig. S1).^22^ After 24 h of attachment, the medium was replaced with Dulbecco’s modified Eagle medium (DMEM), in which calcium ions promoted differentiation of the TIGKs, leading to the formation of a tight monolayer on the collagen IV-coated membrane in Transwells.^23^ Following 4 d of differentiation, the intact monolayer was examined under a microscope (Fig. S2). After confirming monolayer formation, we transferred the Transwell inserts containing TIGK monolayers to the asymmetric gas coculture chamber and placed the entire setup in an anaerobic workstation (Fig. 1b). Unlike typical Transwell assays where both apical and basolateral chambers utilize mammalian cell culture media to support the growth of host cells, we used a specific medium (coculture medium, CCM) introduced in the apical chamber to allow for the maintenance of anaerobes without affecting the viability of TIGKs in the coculture. After placing the asymmetric gas chamber in the anaerobic workstation, the basolateral chamber was stabilized for 3 h with a continuous supply of 10% oxygen at 0.5 standard cubic feet per hour (SCFH). This setup contrasts with the conventional methods, where TIGKs were cocultured with oral anaerobes under a uniform normoxic culture condition in a 5% CO_2_ incubator.^24–26^

We first assessed whether the asymmetric gas chamber could affect the integrity of the TIGK monolayer in the absence of bacterial challenge. We used fluorescein isothiocyanate (FITC)-labeled dextran (molecular weight = 4 000 Da) as a small molecule surrogate to characterize the formation of a tight biological barrier established by the differentiated TIGK monolayer and compared it to the undifferentiated state. 24 h after introducing FITC-dextran in the apical chamber, the fluorescence intensity of the media in the basolateral chamber was quantified to indicate the leakiness of the TIGK monolayer (Fig. 1c). Compared to undifferentiated TIGKs, the diffusion of FITC-dextran through the Transwell under the normoxic condition decreased more than five times, suggesting the formation of a tight monolayer barrier. Moreover, the intactness of the differentiated monolayer under the asymmetric condition remained comparable to that of the normoxic condition. In addition, bright-field imaging of the monolayers showed minimal changes with regards to cell shape or monolayer intactness after 24 h under asymmetric conditions compared to normoxic conditions (Fig. 1d). Notably, the collagen coating affects bright field imaging due to the presence of collagen fibers, which can obscure the fine details of the cells or structures being observed compared to the uncoated surface^24^ (Figs. S1 and S2). The monolayer cells were further dissociated to measure cell viability by flow cytometry. SYTOX Green, a cell viability dye, was applied to quantify the percentage of dead cells. Due to its strong binding affinity to nucleic acids, this dye emits an intense fluorescence in membrane-compromised dead cells. Flow cytometry analysis with SYTOX Green staining indicated no significant increase in cell death under asymmetric conditions compared to normoxic conditions (Fig. 1e, Fig. S3). These results validate that the asymmetric gas chamber maintains the viability and functionality of TIGK monolayers, providing a robust platform for the following studies on characterizing the interactions between anaerobic bacteria and gingival epithelial cells.

### The asymmetric gas coculture system preserves the viability of *Fusobacterium nucleatum*

**Fig. 2:**
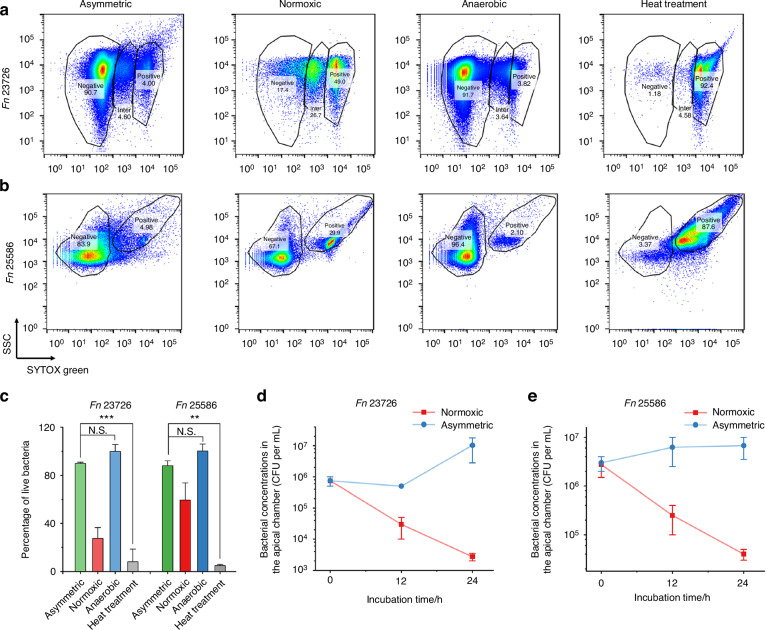
The asymmetric gas setup maintains the viability of *Fn*. Representative flow cytometry results for **a** *Fn* 23726 and **b** *Fn* 25586 under asymmetric gas coculture (with TIGK monolayer), normoxic coculture (with TIGK monolayer), anaerobic condition (in the anaerobic workstation), and dead cell negative control after heat treatment. The MOI was 1. SSC: side scattering. **c** Viability quantification of *Fn* strains 23726/25586 assessed by flow cytometry following different treatment groups, including the asymmetric gas coculture, normoxic coculture, anaerobic culture (positive control), and dead cell negative control after heat treatment (N.S., *P* > 0.05, ** *P* < 0.01, *** *P* < 0.001, *n* = 2 technical replicates, *N* = 3 biological replicates). CFUs of **d** *Fn* 23726 and **e** *Fn* 25586 in the apical chamber under normoxic and asymmetric coculture conditions at 0, 12, and 24 h time points (*n* = 2, *N* = 3). The MOI was 1

In conventional studies of oral anaerobic bacteria-cell interactions, anaerobes such as *F. nucleatum* (*Fn*) are introduced into well plates or Transwells with epithelial cells or other host cell types in a static incubator containing 5% CO_2_ and ambient oxygen levels.^27,28^ We speculate that traditional normoxic conditions can compromise the viability of anaerobes.^29^ In comparison, our asymmetric gas coculture chamber places a monolayer of gingival epithelial cells such as TIGKs as a physiologically relevant barrier between the two chambers, allowing anaerobes to be cultured in the apical chamber under anaerobic conditions. This ensures that the TIGK monolayer limits oxygen diffusion from the basolateral to the apical chamber, replicating the distinct aerobic and anaerobic conditions naturally occurring at the microbial-host interface, particularly within the subgingival pocket where host epithelial cells and anaerobic pathobionts interact.^30^ We hypothesized that utilizing this asymmetric coculture chamber can preserve the viability of *Fn* in the apical chamber compared to the conventional coculture methods under normoxic conditions. To validate this hypothesis, we first tested two commonly used *Fn* model strains (*Fn* 23726 and *Fn* 25586). Instead of using a high multiplicity of infection (MOI) in the literature,^31,32^ which may not be physiologically relevant, we chose an MOI of 1. Bacteria were seeded in the apical chambers of Transwells under asymmetric or normoxic conditions. After 24 h, flow cytometry revealed a significant reduction in bacterial viability under normoxic conditions, with viability decreasing by over 70% for *Fn* 23726. In contrast, the asymmetric gas chamber preserved bacterial viability, comparable to strictly anaerobic conditions (Fig. 2a–c). Similar findings were observed in *Fn* 25586, showing that the asymmetric gas coculture system could maintain the viability of *Fn* 25586 better than the conventional normoxic coculture condition. We reason that oxygen exposure may have compromised bacterial viability under the normoxic condition, affecting how bacteria interact with gingival epithelial cells.^33^ In comparison, the asymmetric gas coculture system offers certain advantages when bacterial viability is critical for characterizing anaerobe-host interactions under physiologically relevant conditions.

In addition to using SYTOX green for measuring bacterial viability, we quantified and compared the temporal changes of colony-forming units (CFUs) of *Fn* 23726 and *Fn* 25586 in the apical chambers of Transwells with differentiated TIGKs under asymmetric or normoxic coculture conditions. At 0, 12, and 24 h post-*Fn* inoculation with an MOI of 1, small aliquots were removed from the apical chamber, serially diluted, and plated on Columbia blood agar plates for CFU quantification. For both *Fn* 23726 and *Fn* 25586, relative to the initial bacterial concentrations, there were 10–100-fold decreases in CFUs under the normoxic coculture at the 24-h endpoint, consistent with the reduced viability of *Fn* strains under the same condition, as evidenced by the SYTOX green staining. However, bacterial concentrations increased over time under the asymmetric coculture condition, suggesting that the viability of *Fn* was maintained (Fig. 2d, e). To confirm if the improved viability was due to the limited oxygen exposure in the apical chamber of the asymmetric coculture system, we performed a control experiment in which *Fn* strains were added to the apical chamber without the differentiated cell monolayer of TIGKs seeded in Transwells. As expected, in the absence of the intact monolayer, a marked reduction in bacterial viability was observed for both strains over time (Fig. S4). Therefore, our findings underscored the essential role of the intact differentiated TIGK monolayer in generating an oxygen barrier between the aerobic basolateral chamber and the anaerobic apical chamber. In addition, the asymmetric gas coculture setup offers an advantage over the conventional coculture system with uniform normoxic conditions to simultaneously support the viability of gingival epithelial cells and anaerobic oral bacteria.

### Differential effects of *Fn* challenge on TIGKs under normoxic and asymmetric conditions

**Fig. 3:**
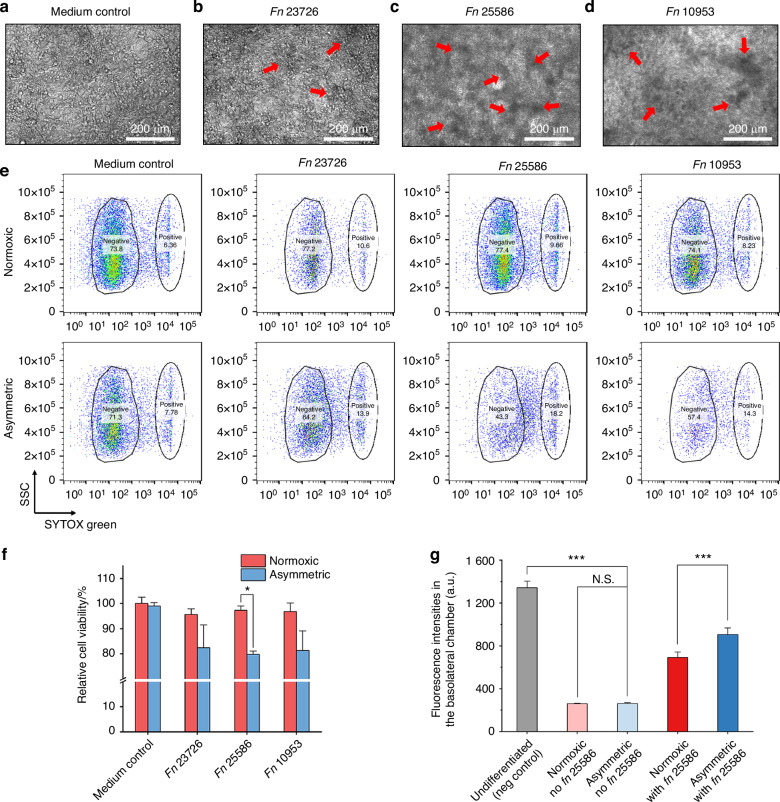
Differential responses of TIGKs challenged by three *Fn* model strains under normoxic and asymmetric gas coculture conditions. Morphological comparison of TIGK monolayers in **a** the medium control (CCM only) and after coculture with **b** *Fn* 23726, **c** *Fn* 25586, and **d** *Fn* 10953 under the asymmetric gas coculture system. The MOI of *Fn* was 1. Red arrows indicate the adherence of bacterial aggregates to the TIGK monolayer. Due to the presence of the collagen fibers, collagen coating can obscure fine details of the cells or structures being observed compared to uncoated surface.^62^ **e** Representative flow cytometry results for TIGKs after coculture with three different *Fn* strains, as assessed by the SYTOX green staining. **f** Quantification of TIGK viability after asymmetric coculture with *Fn* 23726, *Fn* 25586, and *Fn* 10953 based on the above flow cytometry results (0.01 < *P < 0.05, *n* = 2, *N* = 3). **g** Comparison of fluorescence intensities of FITC-dextran in the basolateral chamber at 24 h after adding FITC-dextran to the apical chamber containing TIGK monolayers receiving different treatment conditions. Undifferentiated TIGKs cultured under the normoxic condition served as the negative control due to the lack of an intact monolayer. Differentiated TIGKs with or without *Fn* 25586 challenge under normoxic or asymmetric culture conditions were compared. The background fluorescence of the culture medium was subtracted. The MOI was 1 (N.S., *P* > 0.05, *** *P* < 0.001, *n* = 2, *N* = 3)

Impaired interactions between bacteria and their surrounding epithelium are responsible for bacterial infections.^34–36^ To assess the impact of *Fn* on the host cells in the asymmetric coculture system, we examined the changes in the morphology of the TIGK monolayer directly in the Transwell after coculture with three different *Fn* model strains (*Fn* 23726, *Fn* 25586, and *Fn* 10953) or the medium control for 24 h. In the medium control group, the cell monolayer displayed distinct cell junctions, with uniformly sized cells forming a flat, epithelial-like layer. In contrast, the cell monolayer cocultured with *Fn* exhibited irregular morphology with dense bacterial clusters adhering to the surface (indicated by red arrows) (Fig. 3a–d). Although the same MOI of 1 was used for all three *Fn* model strains, the formation of dense bacterial clusters was particularly pronounced in strains *Fn* 25586 and 10953, likely due to differences at the sub-species level.^37,38^ Nevertheless, these observations align with previous observations on co-aggregation and adhesive properties of *Fn* during interactions with oral epithelial cells.^29^

We further characterized and compared the adverse effects of three different *Fn* model strains (*Fn* 23726, *Fn* 25586, and *Fn* 10953) on the viability of TIGKs under two different coculture conditions. Compared to the medium control (i.e., no bacterium in the apical chamber), coculture with *Fn* 23726, 25586, and 10953 resulted in ~20% reductions in TIGK cell viability under the asymmetric coculture system (Fig. 3e, f). In comparison, there was no noticeable reduction in the viability of TIGKs under normoxic coculture conditions, likely because the viability of *Fn* strains was largely inhibited under the normoxic condition (Fig. 2d, e). Since *Fn* 25586 exhibited a statistically significant differential impact on the viability of TIGKs between asymmetric and normoxic coculture conditions, we further performed the FITC-dextran diffusion assay, in which the diffusion of FITC-dextran inversely correlates with the integrity of the cell monolayer. It was demonstrated that *Fn* 25586 increased monolayer permeability under asymmetric conditions, with fluorescence intensity in the basolateral chamber increasing by 50% over normoxic conditions (Fig. 3g). In summary, these results emphasize the importance of the asymmetric coculture condition in demonstrating the severity of *Fn* infection and the impaired viability and barrier function of oral epithelial cells under physiologically relevant conditions.

### Enhanced *Fn* invasion and elevated pro-inflammatory responses under asymmetric conditions

**Fig. 4:**
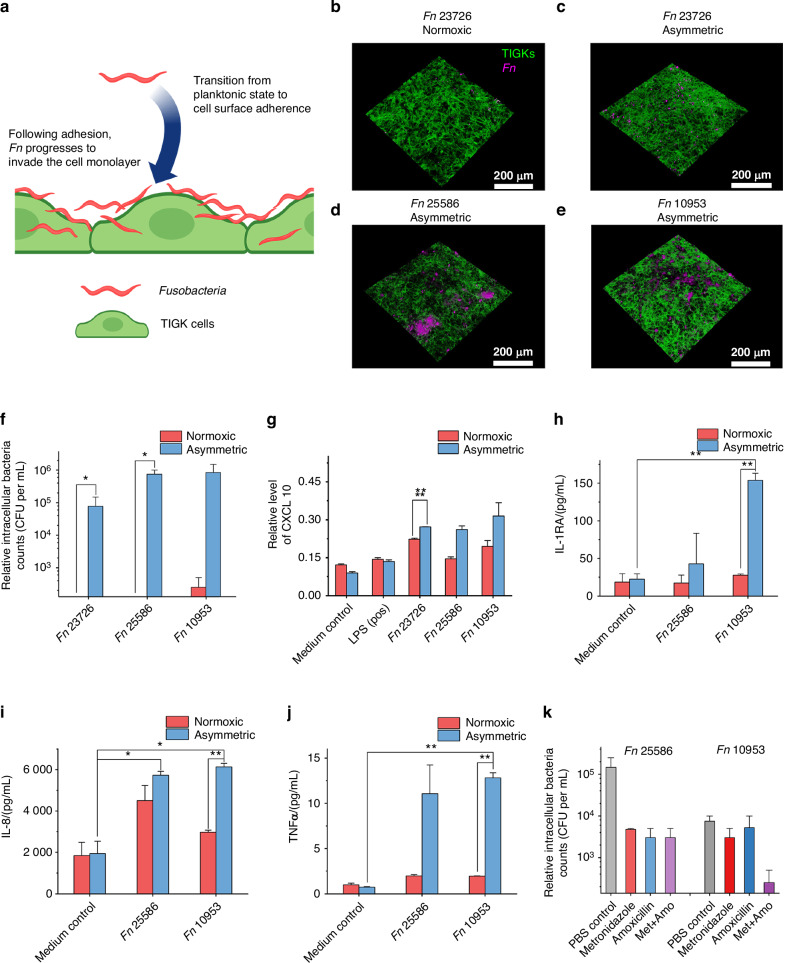
Analysis of *Fn* invasion and its effect on the secretion of proinflammatory factors by cell monolayers. **a** Illustration of *Fn* transitioning from a planktonic state to adherence and invasion to gingival epithelial cells. The figure was created using BioRender. Confocal images showing the interactions between *Fn* and TIGK cell monolayer under normoxic (**b**) and asymmetric coculture conditions, colocalized with Fn strains 23726 (**c**), 25586 (**d**), and 10953 (**e**). *Fn* was prelabeled with a red fluorescence dye DiD, and TIGK cells were post-labeled by Alexa Fluor™ 488 Phalloidin for cytoskeleton. MOI of *Fn* was 50. **f** Differential invasion of three *Fn* strains into the gingival epithelial cells (TIGKs) under normoxic and asymmetric coculture conditions for 24 h. The MOI of *Fn* was 1 (*0.01 < *P* < 0.05; *n* = 2, *N* = 3). **g** Enzyme-linked immunosorbent assay result of CXCL10 (IP-10) expression levels after infection with different *Fn* strains. The value was measured by assessing absorbance at 450 nm, with a reference at 570 nm (*n* = 3, *N* = 3). **h**–**j** Luminex outcome of the difference in expression levels of different pro-inflammatory factors after infection with different *Fn* strains (*0.01 < *P* < 0.05, ** *P* < 0.01; *n* = 1, *N* = 3). **k** Intracellular bacterial counts of *Fn* 25586 and *Fn* 10953 after treatment with amoxicillin, metronidazole, and their combination (*n* = 2, *N* = 3)

*Fn* is well known for its adhesion and invasion of oral epithelial cells.^39^ Adherence to epithelial cells is necessary for colonization, while invasion allows the bacteria to evade immune surveillance and spread to distant organs^25,29,34^ (Fig. 4a). Based on the above findings on the increased *Fn* viability and severity of *Fn* infection (Figs. 2 and 3), we hypothesize that the asymmetric coculture conditions may induce elevated adhesion and invasion of *Fn* to oral gingival epithelial cells. To test the hypothesis, we pre-labeled *Fn* with a red fluorescence dye (DiD) and conducted a short 2-h coculture with TIGKs to minimize the impact of bacterial proliferation (Fig. 4b–e and Fig. S5). After 2 h, TIGK monolayers were extensively washed to remove free bacteria and stained by Alexa Fluor™ 488 Phalloidin specific for the eukaryotic cytoskeleton. Confocal imaging data suggest that *Fn* was more adherent under asymmetric conditions, particularly for *Fn* 25586 and 10953, indicating strain-dependent variations in invasiveness. To further remove unwashed extracellular bacteria to quantify intracellular bacterial loads, TIGKs were dissociated by trypsin and subjected to a cell-impermeable antibiotic, gentamicin, for 2 h. Subsequently, intracellular bacteria were released upon lysis of TIGKs by a surfactant (Triton X-100), and bacterial counts were quantified by plating serially diluted cell lysates on Columbia blood agar plates. As expected, the same trend was observed for each group’s plating result after coculture. Within 2 h of coculture, *Fn* invasion was significantly higher under asymmetric coculture conditions than under the normoxic condition (2 h, an MOI of 50, Fig. S6). *Fn* 25586 exhibited greater invasion into TIGKs than *Fn* 23726, further supporting the differential invasiveness of different *Fn* strains.^40^ Although high MOIs, such as ≥50, were typically used for *Fn* coculture with host cells under the normoxic condition in the literature,^31,32^ we reason that a very high MOI may not be physiologically relevant. Therefore, the monolayers of TIGKs were further infected with three different *Fn* strains with an MOI of 1 for 24 h under normoxic or asymmetric coculture conditions. Afterward, extracellular bacteria were eliminated by gentamicin. Intracellular bacterial counts were quantified by plating serially diluted cell lysates on Columbia blood agar plates. Under the asymmetric coculture condition, the intracellular bacteria counts were approximately 10^5^ times higher than those under the normoxic coculture condition, where intracellular bacteria were almost undetectable (Fig. 4f). This observation was consistent with the increased bacterial proliferation under the asymmetric coculture (Fig. 2). Furthermore, *Fn* 25586 and 10953 exhibited higher intracellular bacterial loads with the same initial MOI of 1 than *Fn* 23726 under the asymmetric coculture condition (Fig. 4f). These findings demonstrate that our asymmetric anaerobic coculture system effectively preserves the invasive characteristics of *Fn* in a physiologically relevant environment, which can be severely affected by normoxic conditions.

Besides serving as a physical barrier, the oral gingival epithelium also provides early immune surveillance to alert the presence of pathogenic bacteria. The direct contact between bacteria and the mucosal surface elicits the secretion of different immune cytokines and chemokines from epithelial cells.^41–44^ Considering the higher invasiveness displayed by *Fn* 25586 and *Fn* 10953 over *Fn* 23726 (Figs. 3 and 4f), we focused on *Fn* 25586 and *Fn* 10953 to evaluate the differential inflammatory responses from TIGKs under normoxic and asymmetric conditions. CXCL10 (IP-10), a key chemokine in the CXC chemokine family, plays a critical role in recruiting different immune cell types and is heavily involved in inflammation and immune regulation. Given the established link between elevated CXCL10 levels and oral bacterial infections of host cells,^39,45^ we quantified the induction of CXCL10 using an enzyme-linked immunosorbent assay. Our results indicated that CXCL10 levels in the bacterial challenge group increased more than twofold compared to the baseline level of the unchallenged group under the asymmetric gas coculture conditions (Fig. 4g). In contrast, under normoxic conditions, the increase in CXCL10 levels in the bacterial challenge group was less than one-fold compared to the unchallenged group. Furthermore, Luminex analysis of additional pro-inflammatory markers revealed significant upregulation of IL-6, IL-8, TNF-α, and IL-1RA (Fig. 4h–j and Fig. S7), consistent with previous studies.^25,28,46^ Of note, the expression of IL-8, a proinflammatory chemokine, has been suggested to be an important regulatory mechanism to induce neutrophil migration into the gingival sulcus.^47^ Taken together, these results highlight the ability of the asymmetric gas coculture system to effectively mimic the proinflammatory responses and cytokine/chemokine secretion typically observed during oral infections, offering an advantage over conventional coculture models for studying bacteria-host interactions in a physiologically relevant environment.

### Antibiotic susceptibility of intracellular *Fn* under asymmetric conditions

To demonstrate the translational potential for applied research, we explored the utility of the asymmetric coculture system for evaluating antibiotic treatment of bacterial infection. The invasion of *Fn* into host cells may contribute to increased resistance to antibiotics, leading to secondary infections.^9^ To assess the efficacy of commonly used antibiotics against intracellular *Fn*, in addition to gentamicin, we selected metronidazole and amoxicillin, two widely prescribed treatments for oral inflammation.^48^ We first demonstrated that extracellular *Fn* in the apical chamber of the asymmetric coculture was effectively inhibited by individual antibiotics, reducing bacterial counts by over three orders of magnitude compared to the untreated group (Fig. S8). We then treated TIGKs with each antibiotic to investigate their effects on intracellular bacteria. Metronidazole and amoxicillin individually exhibited certain inhibitory efficacy against intracellular *Fn* 25586, reducing bacterial load by an order of magnitude, while their combination did not further reduce the bacterial loads (Fig. 4k). In comparison, metronidazole and amoxicillin inhibited intracellular *Fn* 10953, respectively, and their combined use significantly decreased intracellular bacterial load, reducing it to as low as 1% of the original level, which aligns with previous reports.^49–51^ These findings highlight the potential of our asymmetric oral coculture platform as a valuable tool for screening antibiotic susceptibility against intracellular oral pathogenic anaerobes, facilitating the development of targeted therapeutic drugs.

### The asymmetric coculture system supports *Porphyromonas gingivalis*, a highly oxygen-sensitive pathogen

**Fig. 5:**
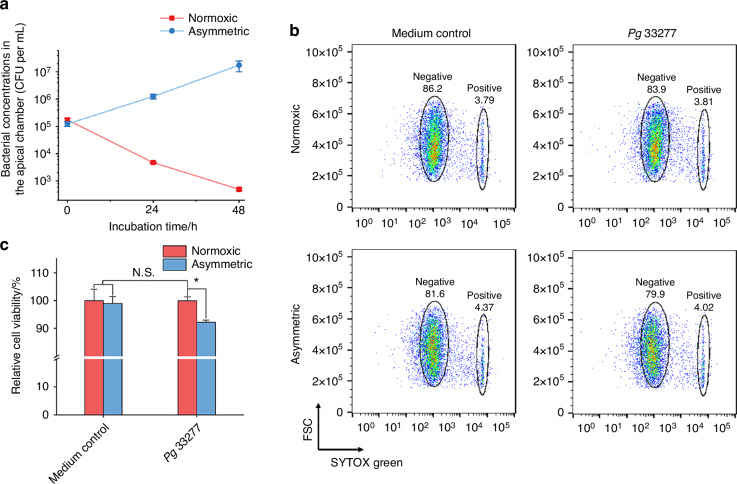
The asymmetric coculture system further enables oxygen-sensitive anaerobe *Pg* to proliferate normally in coculture with TIGK cell monolayers. **a** CFUs of *Pg* 33277 in the apical chamber under normoxic and asymmetric coculture conditions at 0, 24, and 48 h time points (*n* = 2, *N* = 3). The MOI was 1. **b** Representative flow cytometry results for TIGKs after coculture with *Pg* 33277, as assessed by the SYTOX green staining. **c** Quantification of TIGK viability after asymmetric coculture with *Pg* 33277 based on the above flow cytometry results (*0.01 < *P* < 0.05, N.S., *P* > 0.05, *n* = 2, *N* = 3)

Despite being an anaerobe, *Fn* exhibits a relatively high oxygen tolerance.^52^ We next evaluated the ability of our asymmetric coculture system to coculture gingival epithelial cells with more oxygen-sensitive anaerobes, such as *Porphyromonas gingivalis* (*Pg*).^53^ As an oral pathogen, *Pg* is closely related to the progression of periodontitis.^16,54^ We hypothesized that using an oral bacterium with significantly lower oxygen tolerance would rigorously test whether oxygen leakage and diffusion through the cell monolayer into the apical chamber adversely impact bacterial viability. Due to *Pg*’s slower growth rate relative to *Fn*, we inoculated *Pg* into the apical chamber of Transwells containing differentiated TIGK monolayer at the same MOI as *Fn* but extended the coculture duration to 48 h, monitoring bacterial CFUs at 24-h intervals. The *Pg* CFU counts under the conventional normoxic coculture conditions decreased by more than 100 times compared to the initial bacterial concentration (Fig. 5a), underscoring the fact that the conventional coculture setup severely compromised the viability of *Pg* and may not allow for faithful characterization of *Pg-*host cell interactions. Compared to normoxic conditions, however, we showed that asymmetric coculture did not significantly inhibit the normal growth of *Pg*. Its CFU increased approximately tenfold after 24 h and a hundredfold after 48 h, indicating high viability within the asymmetric coculture system. Given that *Pg* is highly sensitive to oxygen and its reproduction is significantly inhibited at low oxygen levels, these findings suggest that the cell monolayer in our system effectively consumes oxygen (consistent with Fig. S4) and demonstrates the monolayer’s role in preventing oxygen from entering the apical chamber, creating an environment conducive to the growth of various anaerobic bacteria. This environment supports not only strains like *Fn*, which can grow at low oxygen concentrations but also strains such as *Pg*, which are sensitive to oxygen.

To assess the impact of *Pg* on gingival epithelial cells under two different growth conditions, we dissociated the cell monolayers, stained them with SYTOX Green, and analyzed cell viability using flow cytometry (Fig. 5b, c). Under normoxic conditions, cell viability was comparable to that of the medium control group, showing no significant difference. In contrast, in the asymmetric coculture group, host cell viability decreased by ~8% compared to the normoxic group, likely attributed to the increased proliferation and pathogenicity of *Pg*.^55–57^

## Discussion

This study highlights the critical need for physiologically relevant in vitro models to study microbial-host interactions, particularly for anaerobic pathogens like *Fusobacterium nucleatum* (*Fn*) and *Porphyromonas gingivalis* (*Pg*). Existing coculture systems, while instrumental, fail to mimic the distinct oxygen environments inherent to the oral environment to maintain the viability of both mammalian cells and anaerobes. The asymmetric gas coculture system developed here addresses these limitations by creating contrasting aerobic and anaerobic conditions that preserve bacterial viability and epithelial cell functionality. This innovation is particularly significant for studying anaerobes, whose growth and pathogenic potential are often compromised in traditional setups. The system’s ability to maintain the integrity of both host and microbial components over extended periods provides a robust platform for investigating complex interactions, including bacterial adhesion, invasion, and immune modulation.

Regarding microbial pathogenesis and host responses, this work demonstrates that the asymmetric coculture model not only sustains bacterial viability but also recapitulates key pathogenic features of *Fn*, such as enhanced invasion and intracellular persistence. These capabilities are pivotal for studying how *Fn* contributes to periodontal disease and its potential systemic implications, including its role in cancers. Moreover, the increased secretion of pro-inflammatory cytokines like CXCL10 and IL-8 under asymmetric conditions underscores the model’s capacity to mimic innate immune responses observed in vivo. By enabling the study of pathogen virulence and host immune dynamics, our system presents exciting opportunities to uncover the molecular and cellular mechanisms underlying microbial pathogenesis and host defense. Others have shown that *Fn* employs several adhesins or outer membrane proteins to facilitate host cell invasion and modulate immune responses. Notably, FadA binds to E-cadherin on host cells, promoting bacterial adherence and invasion, and triggering pro-inflammatory signaling pathways.^58^ Similarly, Fap2 interacts with host cells, contributing to adhesion and invasion processes.^59^ In addition, trimeric autotransporter adhesins such as CbpF, FvcB, FvcC, and FvcD have been identified as key players in host cell interactions.^25^ Future studies could leverage genetic knockout strains lacking individual adhesion genes to elucidate the roles of specific adhesins in host cell binding, invasion, and the activation of innate immune responses. Comparative analyses between knockout mutants and wild-type strains may reveal whether these adhesins function independently or synergistically to drive the invasiveness and pathogenicity of pathobionts under physiologically relevant conditions. Such insights will be pivotal in delineating the molecular determinants of microbial-host interactions and their implications for oral and systemic health.

Beyond advancing fundamental understanding, the asymmetric gas coculture system has significant implications for drug discovery and development. The differential antibiotic susceptibility observed for intracellular *Fn* highlights the importance of testing therapeutics under conditions that closely simulate the physiological microenvironment. The system’s ability to differentiate the effects of antibiotics on extracellular versus intracellular bacteria presents a valuable tool for evaluating targeted therapies. Furthermore, by maintaining the physiological relevance of bacterial and host interactions, this model could streamline the development of novel therapeutics targeting *Fn* and *Pg* for oral infections and related systemic diseases, ultimately bridging the gap between in vitro studies and clinical applications.

Finally, future work could also focus on improving the system’s capabilities by incorporating a flow-based setup that continuously supplies fresh medium to both bacterial and host compartments.^12^ Such an enhancement would allow for extended investigations of microbial-host dynamics over longer time frames, providing insights into chronic interactions and adaptations. Moreover, testing the system with a broader range of cell types, such as immune cells or fibroblasts, and an oral microbial community, would enable the exploration of differential host responses to various microbial species.^13,60^ These advancements could reveal key mechanisms driving oral health and disease and further establish the platform’s utility for personalized medicine and therapeutic screening.

## Materials and methods

### Chemicals

Unless otherwise noted, all chemicals and cell culture broth were purchased from Fisher Scientific International Inc. (Cambridge, MA, USA) and were commercially available of the highest purity or analytical grade. Ultrapure water is provided by the laboratory’s Mili-Q water purification system (MilliporeSigma, Darmstadt, Germany).

### Bacterial strains and growth conditions

*Fusobacterium nucleatum* strains (ATCC 23726, 25586, and 10953) and *Porphyromonas gingivalis* (ATCC 33277) were purchased from the American Type Culture Collection (Manassas, VA, USA). All strains were cultured in liquid Columbia broth (CB) or on CB agar plates containing 5% defibrinated sheep blood (Hemostat laboratories, Dixon, CA, USA) and incubated at 37 °C in an anaerobic chamber (Type A, Coy Laboratories, Grass Lake, MI, USA) containing 5% H_2_, 10% CO_2_, 85% N_2_ (Cryogenic Gases, Ypsilanti, MI).

### Cell strains and culture conditions

The cell line used in this manuscript is the hTERT TIGKs cell line (CRL-3397, ATCC). The base medium used was Dermal Cell Basal Medium (PCS-200-030, ATCC) for culture, to which the components of the Keratinocyte Growth Kit (PCS-200-040, ATCC) were added. The culture atmosphere was 95% Air + 5% CO_2_, and the temperature was 37 °C.

### SYTOX green assay

SYTOX green (S7020, ThermoFisher, Waltham, MA, USA) was added to *Fn* or corresponding cell monolayer apical chamber at a working concentration of 5 μmol/L in phosphate buffer saline (PBS). Fluorescence intensity was then acquired after incubation at room temperature for 10 min. SYTOX green was excited at a wavelength of 488 nm and collected through a bandpass filter from 500 to 550 nm. Transmission images were acquired through a differential interference contrast setting. All the images were acquired under the same image acquisition setting and analyzed by *ImageJ*.

### Transwell collagen coating

All Transwell inserts (76313-906, VWR Inc, Radnor, PA, USA) were precoated with human collagen type IV (C5533, MilliporeSigma) before seeding cells. Specifically, collagen IV was formulated to 1 mg/mL stock solution dissolved in 100 mmol/L acetic acid solution. The collagen stock solution was diluted to 33 μg/mL with ice-cold ultrapure water and mixed well, and 100 μL of diluted solution was aliquoted into each apical chamber of the Transwell inserts. The entire cell plate was incubated at 4 °C for 30 min and then placed in a 37 °C cell culture incubator for 4 h.

### 2D monolayers

2D monolayers were seeded at a density of 200 000 cells per 100 μL in each Transwell insert and incubated for 20 h in the same culture medium used for cell maintenance. Simultaneously, 600 μL of the same medium was added to the basolateral chamber of the Transwell. The following day, the culture medium was replaced with Dulbecco’s modified Eagle’s medium (11965092, ThermoFisher) supplemented with 10% fetal bovine serum (16140071, ThermoFisher) and 100 U/mL Penicillin-Streptomycin (15140122, ThermoFisher). Differentiation was then allowed to proceed for 5 days.

### Asymmetrical coculture setup

A detailed video outlining the assembly steps for the asymmetric coculture chamber is provided as the Supporting Video for this article. Specifically, one day before the experiment, *Fn* was inoculated at a 1:100 ratio into 1 mL of coculture medium (CCM; see Supplementary Table S1 for the specific ingredient list) and grown until the optical density at 600 nm (OD600) reached ~0.4. For *Pg*, the inoculation ratio was increased to 1:5. We transferred a gas-permeable plate (8704000, Coy Laboratories) containing 600 μL of DMEM supplemented with 10% FBS (in the basolateral chamber) into the anaerobic chamber. The plate was secured within the asymmetric coculture chamber using a plate frame with fixing screws. Next, the gas inlet and outlet were connected to the chamber, and magnetic stirring was initiated. Finally, a gas mixture of 10% oxygen, 5% CO_2_, and 85% nitrogen (Cryogenic Gases) was supplied to the basolateral side at a flow rate of 5 SCFH; after 10 s, the flow rate was reduced to 0.5 SCFH to purge residual gas from the system. Meanwhile, the medium in the Transwell inserts was washed with PBS thrice and was replaced with DMEM supplemented with 10% FBS. The inserts were then transferred into the asymmetrical gas system. After 3 h of purge, CCM or the anaerobic bacteria solution (*Fn* or *Pg*) with a multiplicity of infection (MOI) = 1 was used to replace the apical chamber medium. To minimize the impact of hypoxia due to limited diffusion caused by the monolayer’s oxygen consumption, the system was placed atop a magnetic stirrer, ensuring that oxygen is uniformly distributed throughout the basolateral chamber and promotes efficient gas flow. This setup was maintained at 37 °C for 24 h for *Fn* and 48 h for *Pg*.

### Fluorescein isothiocyanate (FITC)-dextran permeability test

FITC-dextran 4 kD (46944, MilliporeSigma) was prepared as a 50 mg/mL working solution. To each apical chamber, 4 μL of the working solution was added to achieve a final 1 mg/mL concentration. The Transwells were then wrapped in aluminum foil to protect them from light and incubated at 37 °C in a cell culture incubator for 2 h. After incubation, 200 μL of the basolateral solution was aspirated and transferred to a black flat bottom 96-well Corning fluorescent plate. Fluorescence intensity was measured with 475 nm excitation and 525 nm emission using a 515 nm filter.

### Laser confocal microscopy of TIGK monolayers coculture with different *Fn* strains

TIGK cells were precultured using the previously described method until day four to establish a mature cell monolayer. *Fn* was pre-labeled with DiD dye (DiIC18(5); 1,1′-dioctadecyl-3,3,3′,3′-tetramethylindodicarbocyanine, 4-chlorobenzenesulfonate salt) at a final concentration of 10 μM and incubated at 37 °C for 30 min. The stained *Fn* was then added to the apical chamber containing the cell monolayer at an MOI of 50 and incubated for 2 h in complete darkness. After coculture, cell monolayers were carefully removed and washed three times with PBS under light-proof conditions. The cell monolayers were then fixed with 4% paraformaldehyde solution at 4 °C for 30 min, followed by three additional PBS washes to eliminate residual paraformaldehyde and prevent interference with subsequent steps. The fixed cell monolayer was carefully cut from the Transwell using a surgical blade and incubated with Alexa Fluor 488 Phalloidin (A12379, Invitrogen), diluted 1:100, at room temperature for 1 h in the dark. The monolayer was washed four times with PBS, each lasting 5 min, to remove any excess phalloidin dye. Following the washes, the sample was placed on a slide and mounted using EverBrite Hardset Mounting Medium (23001, Biotium, Fremont, CA, USA). A coverslip was applied, and the entire sample was stored at room temperature in the dark overnight. Fluorescence images were captured using a Nikon A1 inverted confocal microscope with excitation lasers (640 nm for DiD and 488 nm for Alexa Fluor 488 Phalloidin). The Z-stack function was utilized to acquire three-dimensional images of cell monolayers.

### Human CXCL10 ELISA assay

All apical chamber solutions were analyzed directly by Human CXCL10/IP-10 DuoSet ELISA kit (DY266-05, R&D Systems, Minneapolis, MN, USA). All operations were performed according to the kit protocol instructions. 100 ng/mL lipopolysaccharide (LPS) was applied as the positive control to stimulate the secretion of CXCL10.

### Luminex human cytokine level assay

For sample preparation, the apical chamber solution was centrifuged at 3 000 × *g* three times at 4 °C, with the supernatant collected after each spin to remove cellular debris. The resulting cell culture supernatant was aliquoted into pyrogen-free 0.65 mL snap-top tubes and stored in dry ice. The Human Cytokine Proinflammatory Focused 15-Plex Discovery Assay was conducted by Eve Technologies Corporation (Calgary, AB, Canada). Multiplex analysis was performed using the Luminex™ 200 system (Luminex, Austin, TX, USA). According to the manufacturer’s protocol, fifteen markers were measured simultaneously in samples using the Human-Focused 15-Plex Discovery Assay from Eve Technologies (MilliporeSigma, Burlington, MA, USA). 15 consists of GM-CSF, IFNγ, IL-1β, IL-1Ra, IL-2, IL-4, IL-5, IL-6, IL-8, IL-10, IL-12p40, IL-12p70, IL-13, MCP-1, and TNF-α. Detection sensitivities for these markers ranged from 0.14–5.39 pg/mL for the 13-plex. Sensitivity values for individual analytes are provided in the MilliporeSigma MILLIPLEX^®^ MAP protocol.

### Bacteria level analysis

After the coculture was completed, the apical chamber solution was taken to a serial dilution. Specifically, 90 µL of PBS was added to each well of a 96-well plate, followed by 10 µL of the apical chamber solution to the first well of each row. After thorough mixing, 10 µL was transferred to the second well, and this process was repeated to achieve a tenfold serial dilution across the plate. Bacterial suspensions from each group were then plated on CB blood agar plates, allowing for the determination of the original bacterial count through back-calculation.

For intracellular bacterial counting, following the coculture phase, cells in the Transwell inserts were washed with PBS and digested with 0.25% trypsin for 4 min. The digested cells were resuspended in DMEM containing 50 µg/mL of gentamicin and incubated at 37 °C for 2 h to eliminate any remaining extracellular bacteria. After incubation, the cells were washed twice with PBS and lysed using a solution of 0.1% Triton X-100 and 0.01% SDS in PBS.^27^ The lysates were serially diluted as previously described and plated on CB blood agar plates to quantify the intracellular bacteria. For all other antibiotic treatment groups, the antibiotic concentration was adjusted to 200 µg/mL metronidazole, 200 µg/mL amoxicillin, and the combination of 100 µg/mL metronidazole + 100 µg/mL amoxicillin.^61^

### Statistical analysis

All statistical analyses were performed using GraphPad Prism 9 (San Diego, CA, USA). Data were analyzed using the student *t*-test, a one-way or two-way analysis of variance (ANOVA), followed by the *Bonferroni* test for statistical significance.

## Supplementary information

Supplementary Information

Supporting Video

## Data Availability

All data generated during this study are available within the paper.
