# Supplementary material for: Physiologically relevant coculture model for oral microbial-host interactions
Source: Int J Oral Sci. 2025 May 27;17:42. doi: 10.1038/s41368-025-00365-9 (PMC12117109; doi:10.1038/s41368-025-00365-9)
Supplement: Supplementary file 1 — Supplementary Information [file 41368_2025_365_MOESM1_ESM.docx]

**Supplementary Information**

**Table S1. Co-culture medium (CCM) ingredient list**

| Reagents | Working Concentrations |
| --- | --- |
| Mineral Solutions |  |
| Dipotassium phosphate (K_2_HPO4) | 0.51 mM |
| Potassium diphosphate (KH_2_PO4) | 2.94 mM |
| Potassium chloride (KCl) | 5.37 mM |
| Magnesium sulfate heptahydrate (MgSO_4_·7H_2_O) | 0.41 mM |
| Calcium chloride dihydrate (CaCl_2_·2H_2_O) | 1.26 mM |
| Magnesium dichloride hexahydrate (MgCl_2_·6H_2_O) | 0.49 mM |
| Ammonium sulfate [(NH_4_)_2_SO_4_] | 3.4 mM |
| Sodium chloride | 8 g/L |
| Vitamin Mix |  |
| Biotin | 8.19 μM |
| Folic acid | 4.53 μM |
| Pyridoxine HCl | 48.63 μM |
| Thiamine HCl | 14.82 μM |
| Riboflavin | 13.26 μM |
| Nicotinic acid | 40.61 μM |
| D-Pantothenic acid hemicalcium salt | 20.98 μM |
| Vitamin B12 | 7.37 μM |
| *p*-Aminobenzoic acid (PABA) | 36.46 μM |
| α-Lipoic acid (thioctic acid) | 24.23 μM |
| Trace Metals |  |
| EDTA | 17.1 μM |
| FeSO_4_·7H_2_O | 3.60 μM |
| ZnSO_4_·7H_2_O | 6.26 μM |
| CuSO_4_·7H_2_O | 350 nM |
| CoCl_2_·6H_2_O | 7.57 μM |
| MnSO_4_·H_2_O | 29.58 μM |
| NiCl_2_·6H_2_O | 2.95 μM |
| Glucose | 0.9 g/L |
| Yeast extract | 1.25 g/L |
| Tryptone | 5 g/L |
| Haemin | 2 g/L |
| Bromocresol purple | 1 g/L |
| Sodium bicarbonate | 0.338 g/L |


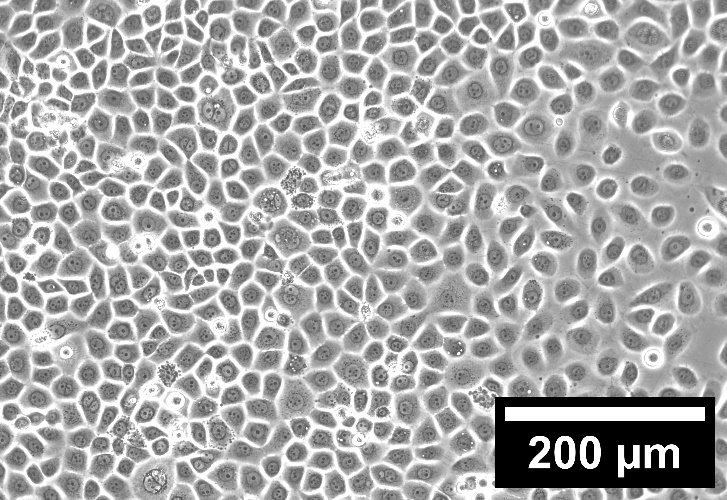


**Figure S1:** Cell morphology of cultured hTERT-Telomerase Immortalized Gingival Keratinocytes (TIGKs) before seeding into Transwell inserts in well plates.


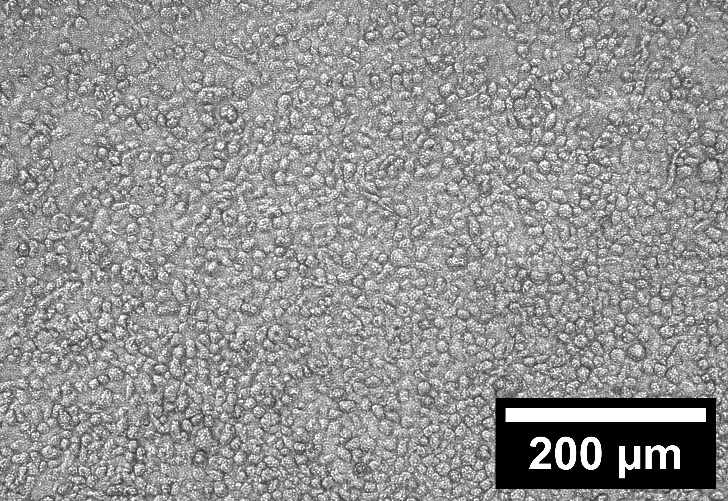


**Figure S2:** Cell morphology of hTERT-TIGK cell monolayer in collagen IV-coated Transwell inserts after 4 d of differentiation within DMEM containing calcium ions. TIGKs form a tight monolayer on the bottom membrane of the Transwell.


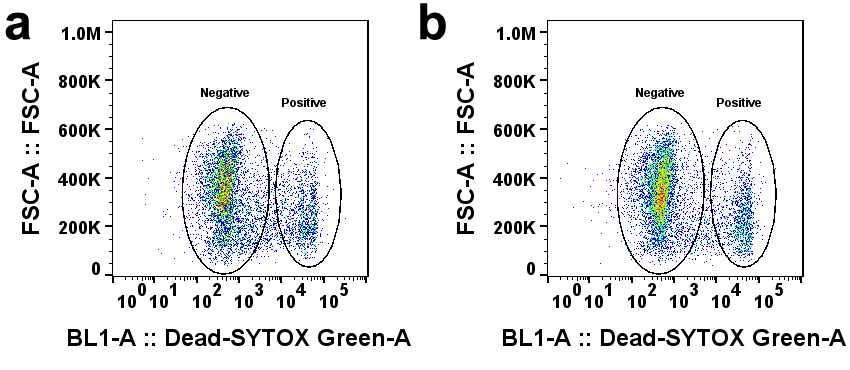


**Figure S3:** Representative flow cytometry results for the viability of TIGKs monolayer cultured under (a) normoxic conditions and (b) asymmetric conditions without bacterial challenge.


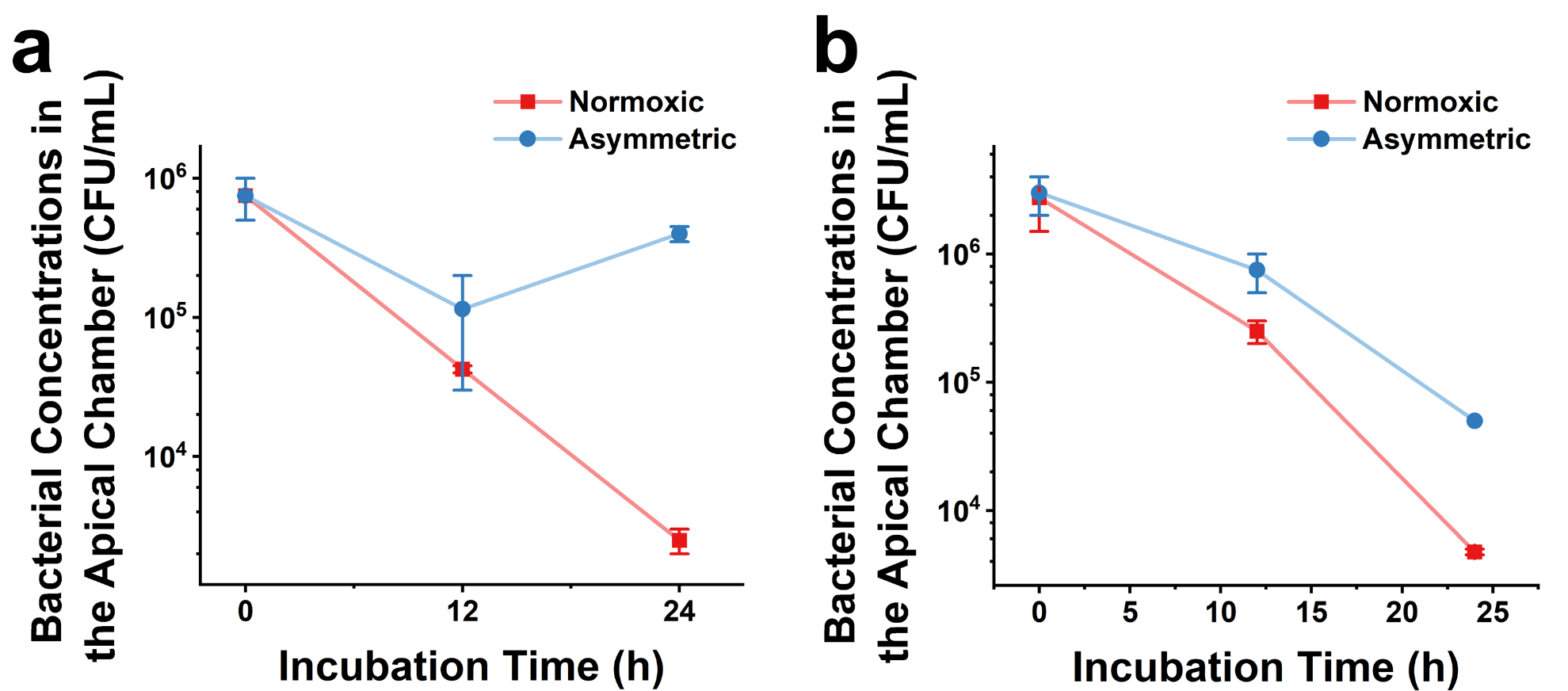


**Figure S4:** (a) Comparison of Fn 23726 concentration changes over 0, 12, and 24 h in the apical chambers without TIGK cell monolayer under normoxic and asymmetric coculture conditions. (b) Comparison of Fn 25586 concentration changes over 0, 12, and 24 h in the apical chambers without TIGK cell monolayer under normoxic and asymmetric coculture conditions (n=2, N=3).


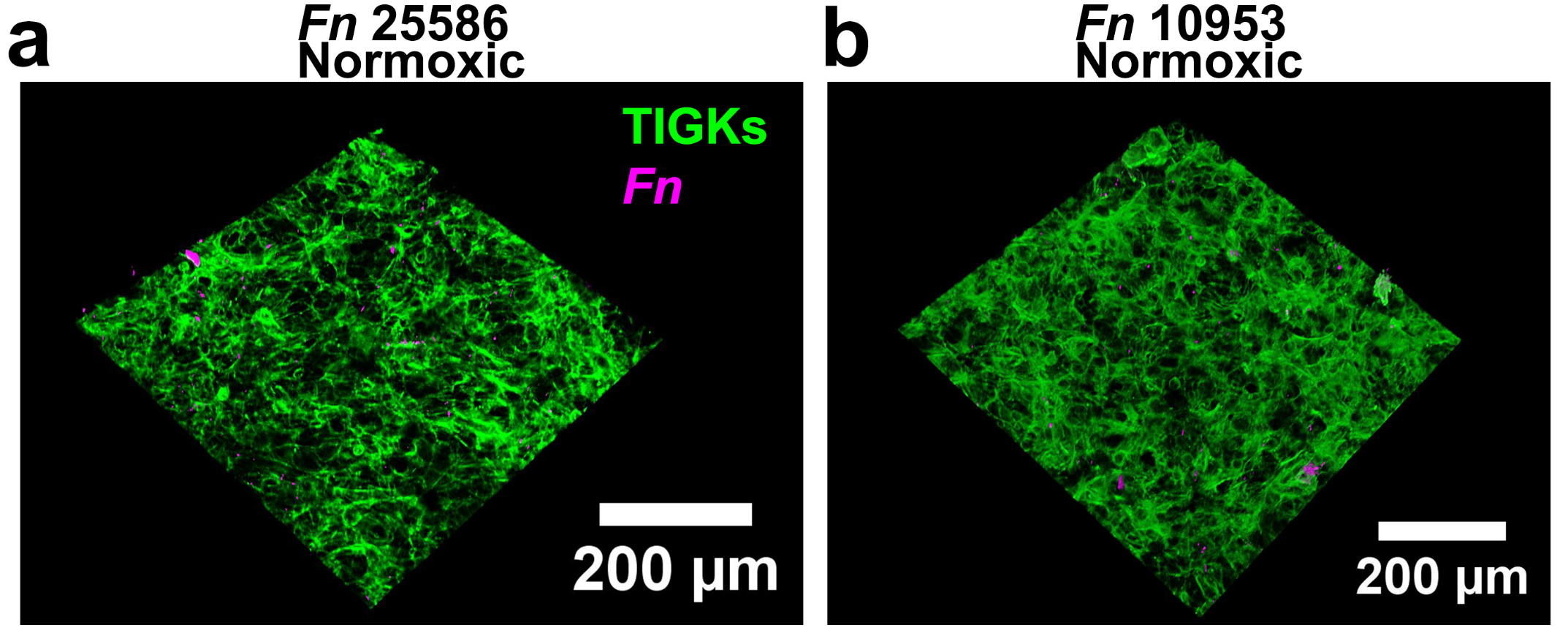


**Figure S5:** Confocal images showing minimal adhesion and invasion of (a) Fn 25586 and (b) Fn 10953 in the TIGK monolayer under normoxic coculture conditions.

**
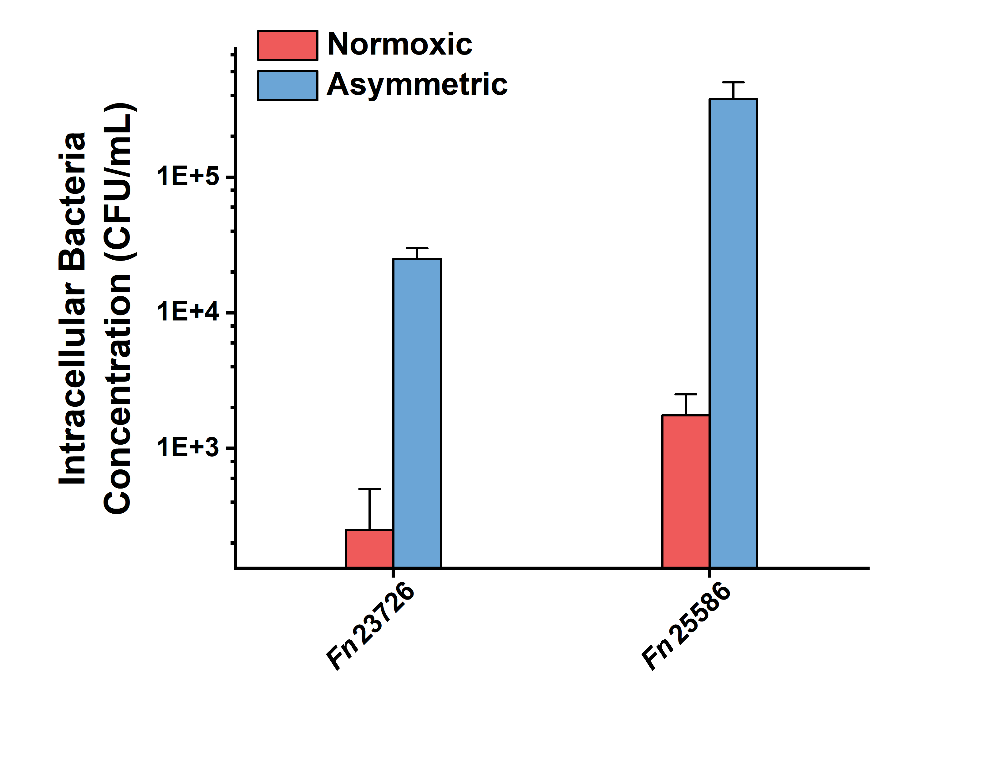
**

**Figure S6:** Analysis of Fn 23726 and 25586 intracellular bacterial concentrations after 2 h of normoxic and asymmetric coculture. Notably, during the 2-h cell invasion under normoxic conditions, the activity of Fn was not fully inhibited due to its brief exposure to oxygen. As a result, under the same starting MOI, the intracellular CFU count was higher than the 24-h normoxic counterparts (n=2, N=3).


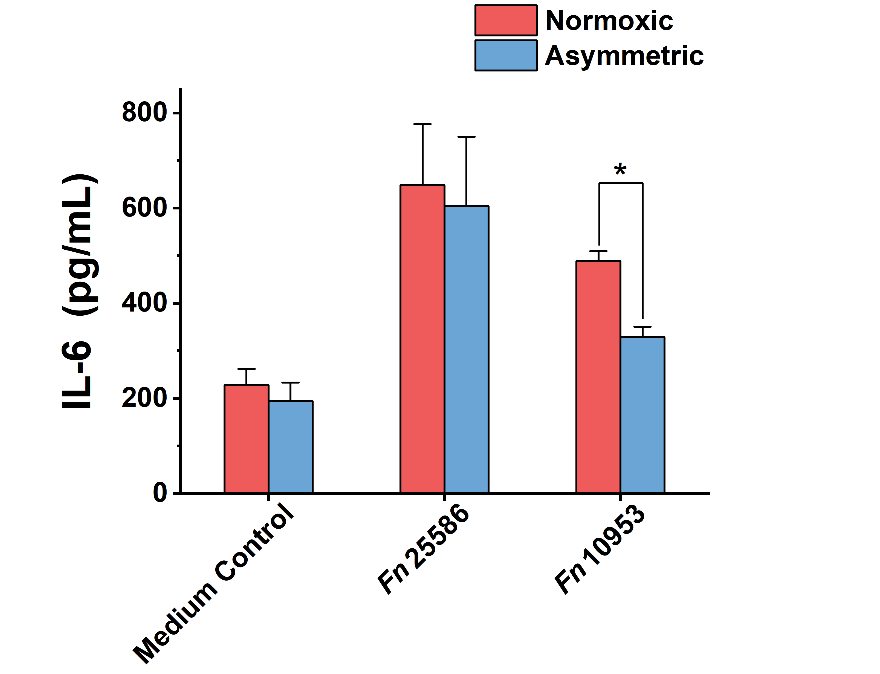


**Figure S7:** Luminex analysis showing differences in IL-6 expression levels in TIGK cell monolayers following infection with various Fn strains (*: 0.01<p<0.05, n=1, N=3).





**Figure S8:** Extracellular concentrations of Fn 25586 and 10953 after treatment with PBS and the same concentrations of gentamicin, amoxicillin, metronidazole, and their combination were used for evaluating intracellular bacteria (n=2, N=3).
